# Switching from intravenous to oral antibiotic therapy in the treatment of infective endocarditis: a case series and literature review of real-world data

**DOI:** 10.1093/jacamr/dlaf032

**Published:** 2025-03-04

**Authors:** Lorenzo Brando Lundgren, Lorenzo Albertini, Anna De Bona, Camilla Tincati, Matteo Augello, Giulia Marchetti

**Affiliations:** Clinic of Infectious Diseases and Tropical Medicine, San Paolo Hospital, ASST Santi Paolo e Carlo, Department of Health Sciences, University of Milan, Milan, Italy; Clinic of Infectious Diseases and Tropical Medicine, San Paolo Hospital, ASST Santi Paolo e Carlo, Department of Health Sciences, University of Milan, Milan, Italy; Clinic of Infectious Diseases and Tropical Medicine, San Paolo Hospital, ASST Santi Paolo e Carlo, Department of Health Sciences, University of Milan, Milan, Italy; Clinic of Infectious Diseases and Tropical Medicine, San Paolo Hospital, ASST Santi Paolo e Carlo, Department of Health Sciences, University of Milan, Milan, Italy; Clinic of Infectious Diseases and Tropical Medicine, San Paolo Hospital, ASST Santi Paolo e Carlo, Department of Health Sciences, University of Milan, Milan, Italy; Clinic of Infectious Diseases and Tropical Medicine, San Paolo Hospital, ASST Santi Paolo e Carlo, Department of Health Sciences, University of Milan, Milan, Italy

## Abstract

**Background:**

The POET trial, along with other studies, indicated that switching from IV to partial oral treatment (POT) in selected infective endocarditis (IE) cases is as effective as the traditional 4–6 weeks of IV therapy. This evidence supported the inclusion of POT in the 2023 European Society of Cardiology (ESC) guidelines, although real-world data remain limited.

**Methods:**

This study retrospectively reviewed IE patients at ASST Santi Paolo e Carlo in Milan, Italy, from January 2018 to July 2022, to identify those who switched to POT. Additionally, a literature review was conducted using PubMed to gather real-world data up to October 2024.

**Results:**

Among 136 IE patients treated at our institution, 9 switched from IV antibiotic therapy to POT. The switch was driven by factors such as lack of venous access, patient preference or improved adherence, and IV antibiotic toxicity. All patients who underwent POT recovered, with no in-hospital or 1 month post-discharge deaths. The literature review uncovered 8 observational studies, 3 case series and 10 case reports, which overall support the effectiveness and safety of POT in selected IE cases, while also highlighting benefits like reduced hospital stays, lower treatment costs and fewer IV-related complications.

**Conclusions:**

In a real-world setting, stepping down to POT after an initial IV regimen proved effective and safe in clinically stable IE patients. This approach offers advantages such as shorter hospital stays, enhanced quality of life and cost savings. Further data are needed to validate these findings and expand the use of oral regimens in broader clinical contexts.

## Introduction

Infective endocarditis (IE) is a life-threatening infection of the inner lining of the heart chambers and valves, or prosthetic material such as valves and intracardiac devices.

Previous international guidelines for the management of IE recommended 4 to 6 weeks of IV antibiotic therapy, resulting in long hospital stays and the need for prolonged IV access, with an ensuing higher risk of hospital-acquired infections.^1,2^

The POET (Partial Oral Treatment of Endocarditis) trial,^3^ together with other randomized controlled trials^4,5^ and observational studies,^6–15^ highlighted that a partial oral treatment (POT) after initial induction with IV standard treatment is non-inferior to prolonged IV treatment. A higher risk of treatment failure with POT strategy was not observed even over a 5 year follow-up in the POET trial.^16^ These findings have been incorporated into the revised 2023 European Society of Cardiology (ESC) endocarditis guidelines.^17^

POT carries several advantages, including shorter hospitalization, reduction of complications associated with IV catheter management, potential cost savings and an improvement in the patient’s quality of life.^18,19^

We herein report our experience in treating IE with partial oral antibiotic therapy, as well as a review of literature evidence from the real world.

## Methods

We retrospectively analysed patients diagnosed with IE at our centre (ASST Santi Paolo e Carlo, Milan, Italy) from January 2018 to July 2022 to identify those switching to POT for any reason. IE cases were identified by two independent clinicians using ICD-10 codes from electronic hospital discharge forms. Demographic and clinical data were retrieved from clinical records. In-hospital mortality and mortality at 1 month post-discharge were also recorded.

A literature research of real-world data up to October 2024 was performed using PubMed. The following terms and similar variations were used in various combinations to search for relevant observational studies, case series and case reports written in English: ‘infective endocarditis’, ‘oral’, ‘switch’, ‘therapy’, ‘oral step down’ and ‘partial oral therapy’.

## Results

### Case series description

During the study period, we observed 136 cases of IE diagnosed by an infectious disease specialist according to the modified Duke’s criteria, of which 9 (6.6%) switched to POT after initial IV antibiotic treatment. The main characteristics of patients who received IV standard treatment or switched to POT are summarized in Tables 1 and 2.

**Table 1.** Demographic and clinical data of patients diagnosed with IE during the study period

Demographic and clinical data of patients diagnosed with IE	IE (*n* = 136)	IV IE (*n *= 127)	POT IE (*n* = 9)
Age, years, median (IQR)	76 (61–81)	76 (62–82)	74 (56–80)
Male sex, *n* (%)	72 (52.9)	67 (52.8)	5 (55.6)
Age-adjusted CCI, median (IQR)	4 (3–7)	5 (4–7)	5 (4–7)
Aetiology, *n* (%)			
MSSA	28 (20.6)	25 (19.7)	3 (33.3)
MRSA	19 (14)	15 (11.8)	4 (44.4)
CoNS	6 (4.4)	6 (4.4)	—
Viridans group streptococci	13 (9.6)	12 (9.4)	1 (11.1)
Ampicillin-sensitive enterococci	17 (12.5)	16 (12.6)	1 (11.1)
Vancomycin-sensitive enterococci	2 (1.4)	2 (1.4)	—
VRE	1 (0.7)	1 (0.7)	—
*Streptococcus bovis*	4 (2.9)	4 (2.9)	—
Other streptococci	7 (5.1)	7 (5.1)	—
HACEK Gram-negative bacteria	1 (0.7)	1 (0.7)	—
Non-HACEK Gram-negative bacteria	7 (5.1)	7 (5.1)	—
Negative blood cultures	31 (22.8)	31 (22.8)	—
IE type, *n* (%)			
NVIE	90 (66.1)	82 (64.6)	8 (88.9)
PVIE	44 (32.5)	44 (36.6)	1 (11.1)
Cardiovascular device	2 (1.4)	1 (0.8)	—
Community acquired	89 (65.4)	87 (68.5)	2 (22.2)
Vegetation dimension, mm, median (IQR)			
Trans-thoracic echocardiography	8 (6–12)	8 (6–12)	12 (10–15)
Trans-oesophageal echocardiography	10 (7–15)	10 (7–15)	12 (8–12)
Laboratory tests, median (IQR)			
WBC (at diagnosis), 10^3^ cells/μL	12 (8–15)	12 (8–15)	11 (10–14)
WBC (at oral switch), 10^3^ cells/μL	—	—	5 (5–7)
WBC (at therapy end), 10^3^ cells/μL	7 (5–10)	8 (5–10)	5 (5–6)
CRP (at diagnosis), mg/L	72 (64–110)	71 (63–110)	76 (33–95)
CRP (at oral switch), mg/L	—	—	13 (8–28)
CRP (at therapy end), mg/L	37 (26–57)	40 (26–57)	5 (5–6)
Complications, *n* (%)	40 (29.4)	34 (26.8)	6 (66.7)
Septic embolization	21 (51.5)	19 (55.6)	2 (33.3)
Pneumonia	4 (10)	2 (5.9)	2 (33.3)
Chordal rupture	6 (15)	5 (14.7)	1 (16.7)
Stroke	1 (2.5)	1 (2.9)	—
Valve regurgitation	2 (5)	1 (2.9)	—
Vertebral osteomyelitis	6 (15)	6 (17.6)	1 (16.7)
Blood culture clearance among cases with positive blood cultures, days, median (IQR)	7 (5–11)	7 (5–11)	4 (3–4)
Treatment, *n* (%)			
Medical	99 (72.8)	91 (71.7)	8 (88.9)
Surgery	37 (27.2)	36 (28.3)	1 (11.1)
Total antibiotic treatment duration, days, median (IQR)	28 (16–42)	26 (14–42)	43 (48–50)
Outcome at hospital discharge, *n* (%)			
Recovery	109 (80.1)	100 (78.7)	9 (100)
Death	27 (19.9)	27 (21.3)	—
Outcome at 1 month post discharge, *n* (%)			
Recovery	98 (72.1)	89 (70.1)	9 (100)
Death	38 (27.9)	38 (29.9)	—

IV IE, IE treated with IV antibiotics; POT IE, IE treated with partial oral antibiotic therapy; CCI, Charlson Comorbidity Index; HACEK, *Haemophilus*, *Aggregatibacter*, *Cardiobacterium*, *Eikenella*, *Kingella*; WBC, white blood cells; CRP, C-reactive protein.

**Table 2.** Demographic and clinical data of the nine IE cases who switched to POT

Demographic and clinical data of the IE cases switched to POT	Patient 1	Patient 2	Patient 3	Patient 4	Patient 5	Patient 6	Patient 7	Patient 8	Patient 9
Age, years	25	82	74	72	80	56	23	79	89
Sex	F	M	M	M	F	M	F	M	F
Risks factor for endocarditis	Mitral valve prolapse	Prosthetic aortic valve	No	No	No	PWID; HIV	No	Previous endocarditis; oral surgery	No
Bacterial isolates	*S. mitis*	*E. faecalis*	MRSA	MSSA	MSSA	MSSA	MRSA	MRSA	MRSA
Localization of endocarditis	Native mitral valve	Prosthetic aortic valve	Native aortic valve	Native aortic valve	Native aortic valve	Native tricuspid valve	Native mitral valve	Native aortic valve	Native aortic valve
Complications	Chordal rupture; septic embolization	No	No	Septic embolization	No	Pneumonia; vertebral osteomyelitis	Pneumonia	No	No
IV antimicrobial treatment	Ampicillin + gentamicin	Ampicillin + ceftriaxone	Vancomycin	Oxacillin	Daptomycin	Cefazolin	Vancomycin	Vancomycin	Vancomycin
IV antimicrobial length (days)	36	23	16	15	10	55	30	20	40
Reason for oral switch	Need for early hospital discharge	Lack of venous access	Lack of venous access	Will of the patient	Will of the patient	Lack of venous access	Lack of venous access	Lack of venous access	IV drug toxicity
Oral antimicrobial treatment	Amoxicillin/clavulanate	Linezolid	Linezolid	Linezolid	Linezolid	Linezolid	Linezolid	Linezolid	Linezolid
POT duration (days)	12	22	26	42	16	11	18	23	≥10

Among the nine patients switched to POT, the median age was 74 (IQR: 56–80) years, and five of them (55.6%) were male. The median age-adjusted Charlson Comorbidity Index (CCI) was 5 (IQR: 4–7). Five patients had no discernible risk factors for IE, one was affected by a mitral valve prolapse, one had a prosthetic aortic valve, one had a history of previous endocarditis and recently underwent oral surgery, while another one was a person living with HIV who injected drugs.

Eight cases (89%) were native valve IE (NVIE), while the other 1 (11%) was a prosthetic valve IE (PVIE); 8 (89%) were left-sided and 1 (11%) a right-sided IE. MRSA was isolated in four cases (44%), MSSA in three cases (33%), and *Enterococcus faecalis* and *Streptococcus mitis* in one case each (11%). At presentation, four patients had one or more IE-related complications, like septic embolization (*n *= 2), pneumonia (*n *= 2), chordal rupture (*n *= 1) and vertebral osteomyelitis (*n *= 1).

All patients were initially treated with IV antibiotics for a median of 23 (IQR: 16–36) days before switching to POT, which was continued for a median of 18 (IQR: 12–23) days, accounting for a total median antibiotic therapy duration of 48 (IQR: 43–50) days. In all cases, patients were apyretic and haemodynamically stable at time of switch to oral therapy; bacteraemia was already cleared and inflammatory markers were reduced over time. Right before switching, CT scans were performed to rule out septic embolization, as was echocardiography to exclude local disease progression. Reasons for switching to POT were: lack of venous access (5 patients, 56%), improvement of patients’ adherence or patients’ wish (2 cases, 22%), previous IV antibiotic toxicity (1 case, 11%), or need for early hospital discharge (1 case, 11%). In eight patients (89%) the oral antibiotic was linezolid and in 1 case (11%) it was amoxicillin/clavulanate.

Oral antibiotics were well tolerated as no adverse events were registered. Clinical cure was reached in all patients at the time of discharge and no IE relapses or deaths were observed during hospitalization and 1 month post-discharge.

### Literature review of real-world data

Ten observational studies, 3 case series and 10 case reports were identified (Tables 3 and 4).

**Table 3.** Observational studies assessing the switch to partial oral antibiotic therapy in the treatment of IE

Study (year)	Study design (country)	Population	Sample size	Oral antibiotics	Follow-up	Primary outcome	Key results	Conclusions	Limitations
Dworkin *et al.* (1989)^10^	Pilot non-randomized clinical trial (USA)	Definite right-side endocarditis in PWID	14	Ciprofloxacin and rifampicin	28 days	Mortality and relapse	POT was safe and effective (95%)	POT is safe and feasible in PWID with *Staphylococcus aureus* right-sided IE	Small sample size; no comparative group
Colli *et al.* (2007)^9^	Retrospective observational study (Italy)	Definite left-side IE after cardiac surgery	14	Linezolid	Median follow-up: 21 months	Mortality and relapse	All patients were cured; no relapse during follow-up	Combination of POT and surgery is safe and feasible	Small sample size; no comparative group
Demonchy *et al.* (2011)^8^	Retrospective observational study (France)	Definite or possible IE	66 [POT (19) versus IV (47)]	Fluoroquinolones (9), amoxicillin (7), linezolid (3)	Median follow-up: 90 days	Overall mortality	No association between oral antibiotic use and increased mortality	POT may be safe in selected patients	No formal protocol guiding the switch to oral therapy
Mzabi *et al.* (2016)^7^	Retrospective observational study (France)	Definite or possible IE	426 [POT (214) versus IV (212)]	Amoxicillin (109), fluoroquinolones (32), other (9)	Median follow-up: 140 days	Mortality, relapse and reinfection	No significant differences in POT and IV groups	POT is feasible in less severely ill patients	Timing of the oral switch was highly variable; selection bias
Tissot-Dupont *et al.* (2019)^6^	Retrospective observational study (France)	Definite *S. aureus* IE	341 [POT (171) versus IV (170)]	Cotrimoxazole	Median follow-up: 166 days	Overall mortality during hospital stay, 30 day mortality, 90 day mortality	POT group had shorter hospital stay and lower in-hospital and 30 day mortality	Rapid switch to POT reduced the length of hospital stay and mortality rate	MRSA represented only 10% of the study population
Marks *et al.* (2020)^11^	Retrospective observational study (USA)	Invasive infections in PWID including endocarditis	293 (166 IE) [partial IV (67) versus POT (83) versus total IV (143)]	N/A	N/A	All-cause 90 day readmission	POT had similar readmission rates compared with IV	POT reduced 90 day readmission rate compared with partial IV	Results may not be generalizable to other institutions
Lewis *et al.* (2022)^14^	Prospective observational study (USA)	Invasive infections in PWID including endocarditis	166 (68 IE) [IV (32) versus POT (36)]	N/A	Median follow-up: 90 days	90 days all cause readmission, 90 days mortality, 90 day emergency department admission, microbiological failure and death	POT had similar readmission and mortality rates compared with IV	POT and adherence are feasible in PWID	Results may not be generalizable to other institutions
Wildenthal *et al.* (2022)^15^	Retrospective observational study (USA)	Complicated *S. aureus* bacteraemia and incident IE in PWID	238 (154 IE) [partial IV (22) versus POT (36) versus total IV (90)]	Amoxicillin/clavulanate, cefalexin, cefadroxil, dicloxacillin, clindamycin, doxycycline, ciprofloxacin, linezolid, rifampin, cotrimoxazole	Median follow-up: 90 days	Microbiological treatment failure at 90 days and readmission	POT and total IV had similar microbiological failure rates and readmission rates	POT is feasible in PWID declining standard of care, especially after ≥10 days IV	Results may not be generalizable to other institutions
Freling *et al.* (2023)^12^	Retrospective observational study (USA)	Definite or possible IE	257 [POT (46) versus IV (211)]	Linezolid (30), penicillin (8), fluoroquinolone (6), cotrimoxazole (2)	Median follow-up: 90 days	Clinical cure at 90 days	No differences in 90 day clinical success	Similar outcomes of real-world use of POT versus IV	Possibility of missing follow-up
Camp *et al.* (2024)^13^	Retrospective observational study (Germany)	Definite or possible IE	178 [POT (30) versus IV (148)]	Amoxicillin (13), fluoroquinolones (9), cotrimoxazole (5), others (3)	Minimum 90 days after IE diagnosis	Mortality and infection-related complications	POT group had lower mortality (17%) compared with IV group (41%)	POT is feasible and safe with careful patient selection	Selection bias in POT group

**Table 4.** Case series/reports assessing the switch to partial oral antibiotic therapy in the treatment of IE

Study (year)	Patients (*n*)	Age (years)	Male sex, *n*, (%)	CCI (median) or comorbidities	Surgery (%)	Type of IE	Complications, *n* (%)	Aetiology (*n*)	IV therapy (days)	POT (days)	Total therapy (days)	POT antibiotics (*n*)	Cure and follow-up	Reason for POT switch
Miller *et al.* (2022)^20^	11	Median: 32	7 (63.6)	0	5 (55)	NVIE (4), PVIE (7)	7 (77)	MSSA (3), viridans group streptococci (3), *E. faecalis* (2), negative bacterial culture (2), *Candida albicans* (1)	Median: 13 (IQR: 4–22)	Median: 23 (IQR: 12–38)	N/A	Linezolid (6), levofloxacin (2), moxifloxacin (2), rifampicin (2), dicloxacillin (1), amoxicillin (4), amoxicillin/clavulanate (1), fluconazole (1)	Cured at discharge and negative FU (3 months)	Patients’ desire, IV management issues
Parker and Fossieck (1980)^21^	35	Median: 31	35 (100)	Unknown; 29 (93%) were PWID	0	NVIE (35)	0	MSSA (35)	Mean: 16.4 (SD: 4–33)	Mean: 26 (SD: 14–58)	Mean: 42.4 (SD: 34–69)	Dicloxacillin, oxacillin, clindamycin, penicillin V	Cured at discharge	Unknown
Attanasio *et al.* (2020)^22^	5	Median: 61	4 (80)	2	0	PVIE (5)	4 (80)	*E. faecalis* (5)	Median: 28 (IQR: 18–41)	Median: 120 (IQR: 78–174)	N/A	Amoxicillin/clavulanate (5), cefditoren (5)	Cured at discharge and negative FU (6 months)	Unknown
Alsaeed *et al.* (2023)^23^	1	17	1 (100)	0	0	NVIE	1	MSSA	17	51	68	Linezolid, rifampicin	Cured at discharge and negative FU (6 months)	Patient’s family decision
Archuleta *et al.* (2004)^24^	1	64	1 (100)	5 (HIV/HCV coinfection and renal transplant recipient)	0	NVIE	0	VRE	17	25	42	Linezolid	Cured at discharge and negative FU (6 months)	Unknown
Babcock *et al.* (2001)^25^	1	34	0 (0)	4 (Down syndrome, end stage renal disease with haemodialysis, congenital cyanotic heart disease)	0	NVIE	0	VRE	12	42	54	Linezolid	Cured at discharge and negative FU (3 months)	IV management issues
Bruun *et al.* (2011)^32^	1	52	1 (100)	1	1	NVIE	1	*Streptococcus anginosus*	0	42	42	Phenoxymethylpenicillin, roxithromycin, amoxicillin/clavulanate	Cured at discharge	Postponed diagnosis
Colkesen (2021)^26^	1	83	0 (0)	4	0	NVIE	0	MSSA	14	28	42	Moxifloxacin	Cured at discharge and negative FU (6 months)	Unknown
Guntheroth (1984)^27^	1	23	0 (0)	2 (cognitive deficit, ventricular septal defect)	0	NVIE	0	*Streptococcus sanguinis*	0	30	30	Amoxicillin	Cured at discharge and negative FU (12 months)	Patient and family’s wish
Mahmoud *et al.* (2020)^28^	1	65	1 (100)	4 (chronic kidney disease, ischaemic and valvular heart disease)	0	PVIE	1	*E. faecalis*	0	365	365	Amoxicillin/clavulanate	Cured at discharge and negative FU (12 months)	Patient’s wish
Nathani *et al.* (2005)^29^	1	28	0 (0)	1 (severe asthma)	1	NVIE	0	MRSA	0	28	28	Linezolid	Cured at discharge	IV management issues
Ng *et al.* (2005)^30^	1	37	1 (100)	0 (rheumatic heart disease)	0	NVIE	0	*S. mitis*	19	28	47	Linezolid	Cured at discharge and negative FU (1 month)	IV management issues
Ravindran *et al*. (2003)^31^	1	74	0 (0)	3 (mitral valve prolapse, ventricular arrests history, enterocutaneous fistula)	1	NVIE	1	*Staphylococcus epidermidis*	31	>56	>87	Linezolid	Death due to other cause	IV management issues, vancomycin toxicity

VSG, viridans streptococci group; VRE, vancomycin-resistant *E. faecium*; FU, follow-up.

Among observational studies, those from Tissot-Dupont *et al.*,^6^ Mzabi *et al.*^7^ and Freling *et al.*^12^ emphasized that switching to oral antibiotics significantly reduces hospital stay without negatively affecting clinical outcomes.

In particular, Tissot-Dupont *et al.*^6^ found that patients who switched to oral antibiotics had a shorter hospital stay and lower short-term mortality rates compared with those continuing the IV regimen, although the 90 day mortality rate remained similar. These findings suggest that oral antibiotics may improve patient comfort and reduce healthcare costs without increasing the risk of adverse outcomes.

Studies by Mzabi *et al.*^7^ and Freling *et al.*^12^ showed no significant differences in relapse, reinfection or mortality—both in the short and the long term—between patients receiving oral versus IV therapy, thus suggesting that oral antibiotics may be as effective as IV treatment for certain patients, particularly those with less severe disease or favourable clinical conditions.

A common limitation across these studies is the potential selection bias inherent in deciding which patients to switch to oral antibiotics. For instance, Tissot-Dupont *et al.*^6^ and Camp *et al.*^13^ highlighted that patients selected for oral therapy generally had milder forms of IE with smaller vegetations, which may have skewed the results in favour of oral regimens. This emphasizes the need for rigorous criteria when selecting patients for oral therapy, as outcomes may not be as favourable for those with more severe infections or complications. However, some studies, like that of Demonchy *et al.*,^8^ explored the use of oral antibiotics in patients with more complicated forms of IE, such as those with haemodynamic instability, embolic events or abscesses. Despite the severity of these cases, no increase in mortality was observed in the oral therapy group, suggesting that oral regimens might still be viable in selected complex cases.

Dworkin *et al.*,^10^ Marks *et al.*,^11^ Lewis *et al.*^14^ and Wildenthal *et al.*^15^ focused on people who inject drugs (PWID), a population in which adherence to long-term IV therapy can be particularly challenging. The long-term success of oral therapy in these patients suggests this strategy may be an essential tool for improving adherence and reducing hospitalizations in this high-risk group.

Furthermore, Colli *et al.*^9^ demonstrated that early switching to oral linezolid post-cardiac surgery was effective in preventing relapse and infection recurrence, indicating that oral antibiotics may also be a valuable option in the postoperative management of IE.

Lastly, the study by Freling *et al.*^12^ showed that oral antibiotics can reduce adverse events associated with prolonged IV therapy, such as acute kidney injury and IV catheter-related complications. This is a relevant issue in the management of IE, as prolonged IV antibiotic therapy increases the risk of such complications, which can negatively affect patient outcomes and quality of life.

Overall, data from observational studies suggest that oral antibiotic therapy can be a safe and effective option for managing IE, potentially reducing hospital stays, healthcare costs and IV catheter-related complications, although careful patient selection is crucial to maximize the benefits of this approach.

POT has also been shown to be an effective strategy in several case reports and case series, which provided a nuanced perspective on diverse clinical situations and therapeutic approaches (Table 4).^20–32^

## Discussion

Based on the long-term data from the POET trial,^3^ the 2023 ESC endocarditis guidelines recommended partial oral antibiotic therapy as a switching strategy in stable patients with IE.^17^ POT has also been proven as an effective treatment strategy for clinically stable patients in real-life settings.

In the present case series, we highlight the successful treatment of nine IE cases with partial oral antibiotic therapy. Only a small percentage (6.6%) of patients were switched to POT in our centre within the study time period despite the availability of high-quality data from randomized controlled trials (RCTs) demonstrating its efficacy and safety. This is likely because the oral approach was not yet recommended by international guidelines at the time of treatment. Moreover, in the few cases where the switch occurred, the decision appears to have been primarily driven by practical needs, such as the lack of adequate venous access and the patient’s intolerance to the hospital stay, which would have jeopardized effective treatment.

In keeping with previous literature, linezolid and amoxicillin/clavulanate were the most frequently used antibiotics for POT in our cases. Despite oral antibiotic monotherapy having been shown to be effective in several real-world studies,^6,7,9,12,13,15^ as well as in our case series, in the wake of the POET trial design, the revised ESC guidelines suggest combination oral regimens consisting of two antibiotics from different drug classes with different antimicrobial mechanisms of action and different metabolization processes to ensure adequate concentrations of at least one antibiotic, e.g. in the case of reduced gastrointestinal uptake or fast metabolization of one drug. Nonetheless, there is currently no evidence that combination oral antibiotic therapy is superior to monotherapy. This issue ought to be addressed in future RCTs, as oral combination therapy may be associated with increased toxicity and healthcare costs and potential antagonism, as well as decreased patient adherence.

Several antibiotics—like linezolid, amoxicillin, rifampin, moxifloxacin and fusidic acid—have excellent oral bioavailability and tissue penetration, and are thus optimal candidates for POT. However, intact gastrointestinal absorption function and normal drug metabolization processes are required to guarantee adequate plasma concentrations and thus the efficacy of oral therapy. Therefore, outpatient follow-up programmes including therapeutic drug monitoring (TDM) should be implemented in the management of such patients, in order to ascertain adequate plasma concentrations while limiting the risks of toxicity.

Notably, our case series, together with some other case reports/series, suggest that POT may also be an effective strategy in IE sustained by MDR bacteria such as MRSA or VRE, thanks to the activity of some oral antibiotics against such pathogens. Likewise, our cases and a previous study^8^ suggest that POT may be also considered in selected complicated cases, even though this aspect should be more specifically addressed in future research.

The adoption of POT, although supported by guidelines, may face various barriers in clinical practice, including the following: the need for accurate patient selection to minimize the risk of treatment failure, as those with severe complications or conditions affecting oral absorption may not be ideal candidates; pharmacokinetic challenges, such as variable drug absorption and drug–drug interactions that may compromise efficacy; patient adherence and the need for close monitoring that complicate outpatient management; microbiological factors, like polymicrobial infections or resistant bacteria, which can limit the effective oral treatment options; and clinician confidence and established local IV protocols that may hinder the adoption of evidence-based recommendations.

Although limited in size and retrospective in nature, the present case series adds to the real-world data supporting POT as a treatment for selected patients not suitable for standard IV therapy or who may benefit from early discharge. Future prospective studies and standardized protocols for switching to oral therapy are needed to further validate these findings and expand the use of oral regimens in broader clinical contexts.
